# A Case of Squamous Cell Carcinoma of the Breast Occurring in Benign Phyllodes Tumor

**DOI:** 10.7759/cureus.73943

**Published:** 2024-11-18

**Authors:** Kumiko Hayashi, Goshi Oda, Tsuyoshi Nakagawa, Tomoyuki Fujioka, Iichiroh Onishi

**Affiliations:** 1 Department of Breast Surgery, Institute of Science Tokyo Hospital, Tokyo, JPN; 2 Department of Breast Surgery, Dokkyo Medical University Hospital, Tochigi, JPN; 3 Department of Artificial Intelligence Radiology, Institute of Science Tokyo Hospital, Tokyo, JPN; 4 Division of Surgical Pathology, Institute of Science Tokyo Hospital, Tokyo, JPN

**Keywords:** benign breast tumor, breast cancer pathology, breast surgery, phyllodes tumour, squamous cell carcinoma (scc)

## Abstract

A 49-year-old woman visited our hospital after noticing an enlarged left breast mass. After a biopsy, she was diagnosed with fibroadenoma and underwent tumor resection. Postoperative pathology revealed squamous cell carcinoma (SCC) within a benign phyllodes tumor. Following additional resection, adjuvant chemotherapy was administered in accordance with the treatment for triple-negative breast cancer, and the patient has since progressed without recurrence for four years. The occurrence of SCC within a phyllodes tumor is extremely rare, and we report this case through a review of the literature.

## Introduction

Primary squamous cell carcinoma (SCC) of the breast is classified as a special type of breast cancer. Its frequency among all breast cancers is less than 0.1% in Japan [1], making it an extremely rare disease. Phyllodes tumors are also rare, accounting for 0.3%-0.5% of all breast tumors [2]; a case of breast cancer within a phyllodes tumor is therefore extremely rare.

In terms of clinical prognosis, SCC of the breast is widely reported to have a poor prognosis due to rapid growth. The treatment of SCC is controversial, but the same treatment for the more common types of breast cancer usually seems to be selected.

Here, we report a rare case of SCC within a benign phyllodes tumor. Although it was expected that a benign tumor would be more likely from imaging studies and biopsy results, surgical excision was operated due to the clinical course of rapid growth of the tumor. The patient had a favorable outcome due to early intervention that included surgery, radiation therapy, and chemotherapy.

## Case presentation

**Figure 1:**
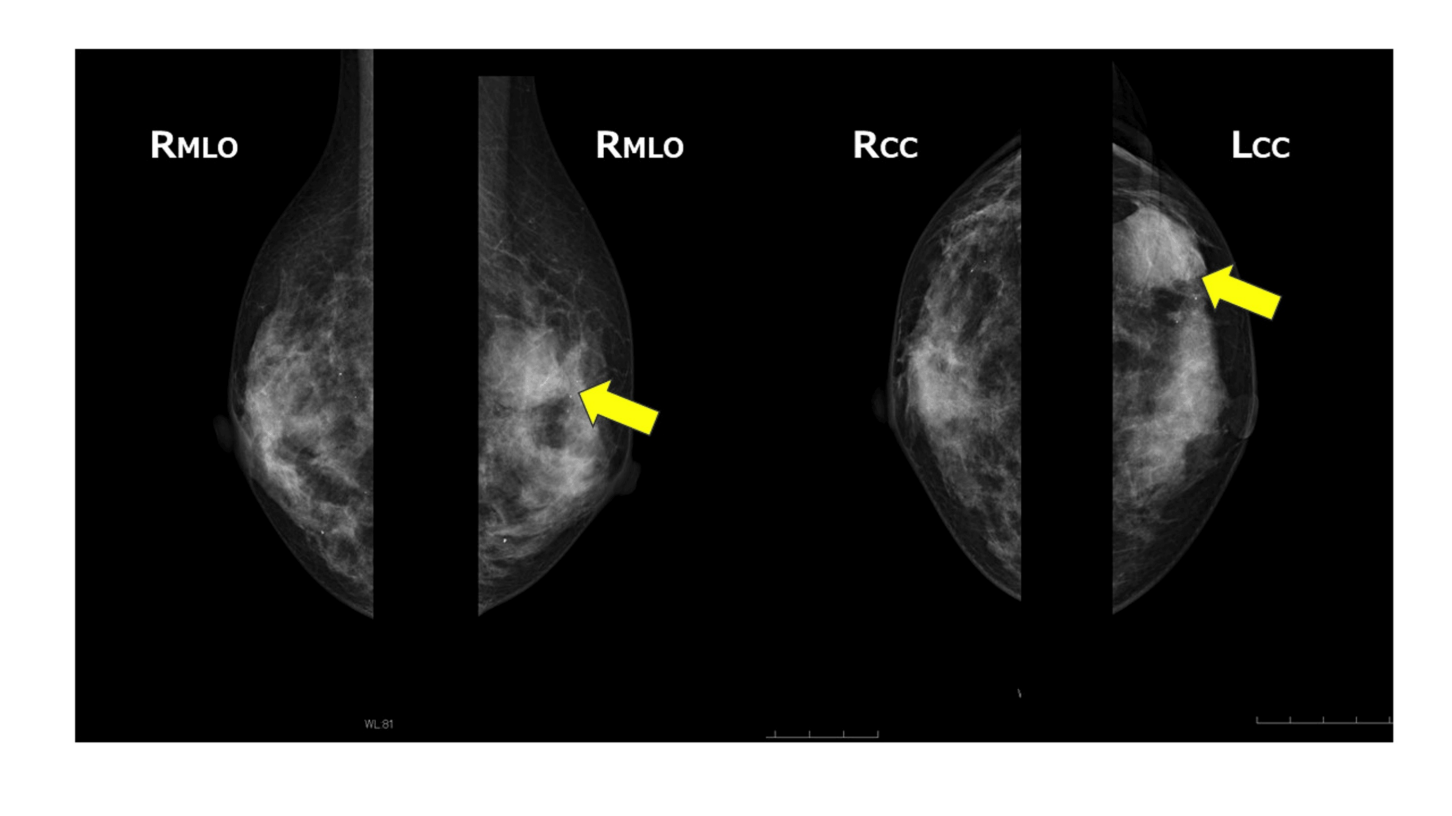
Mammography findings in this case. Left medio-lateral oblique and cranio-caudal mammograms demonstrate a 31mm well-defined hyperdense mass in the upper outer breast (yellow arrows).

**Figure 2:**
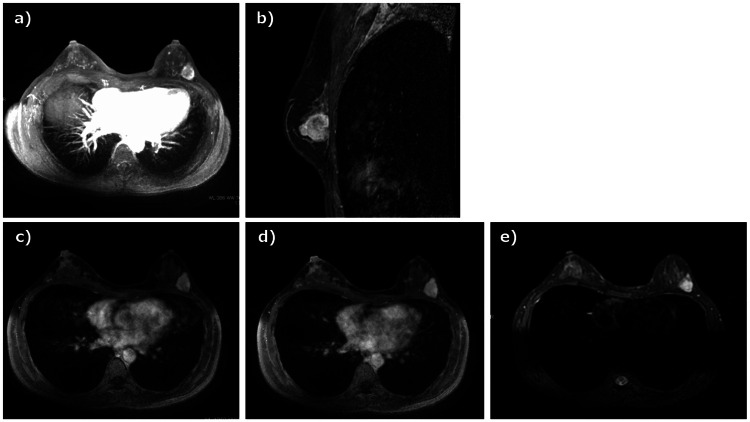
MRI findings in this case. MRI shows a 31mm well-defined oval mass that exhibits contrast enhancement with a heterogeneous fast-persistent pattern in the upper outer area of the left breast. (a) Maximum intensity projection image. (b) Sagittal section image. (c) Initial phase of dynamic contrast-enhanced MRI. (d) Delayed phase of dynamic contrast-enhanced MRI. (e) Fat-suppressed T2-weighted image.

**Figure 3:**
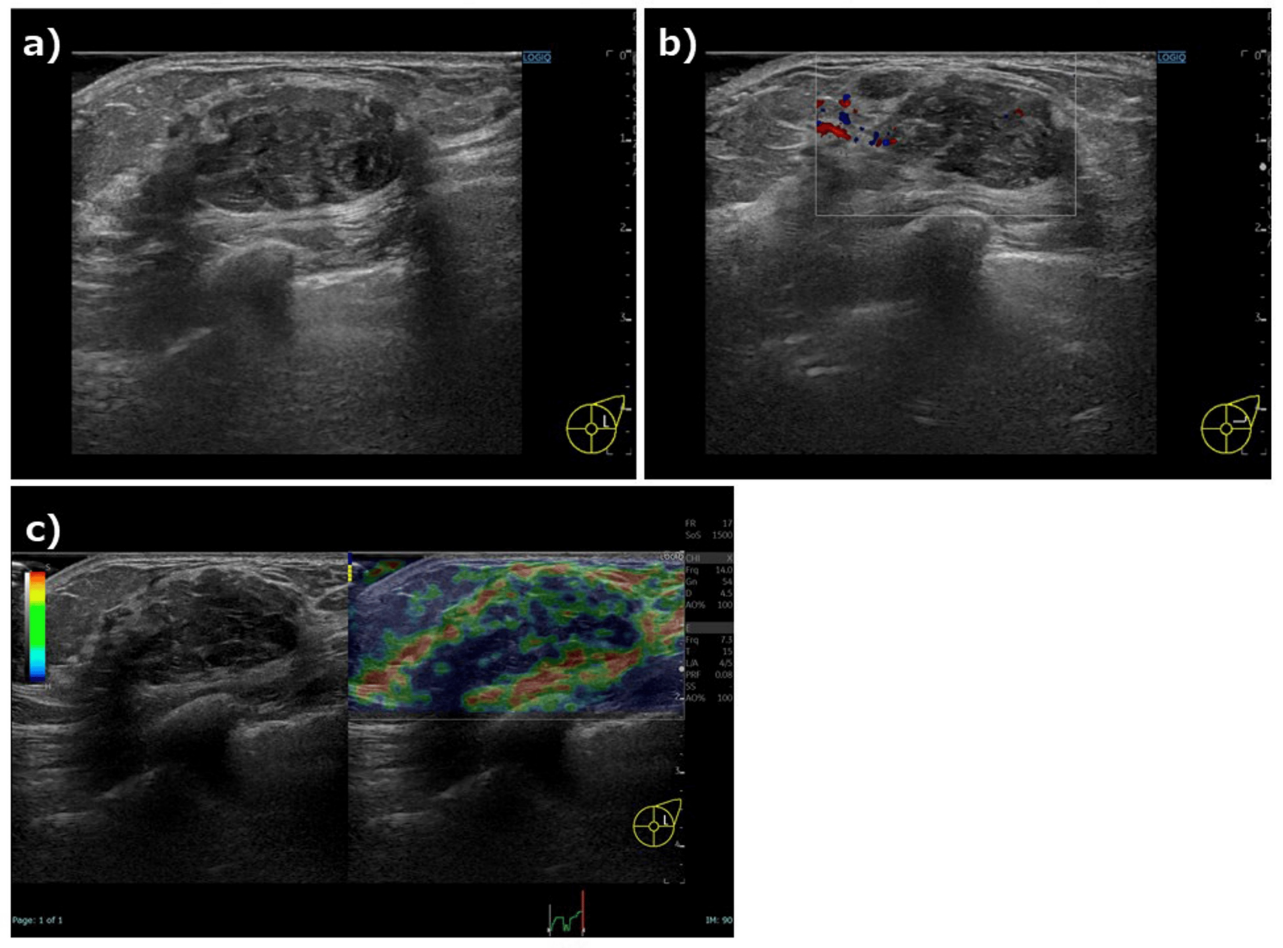
Ultrasonography findings in this case. Ultrasonography reveals a 26-mm well-defined hypoechoic mass in the upper outer area of the left breast (a). Power doppler ultrasonography shows internal blood flow within the mass (b), and elastography shows decreased strain (c).

**Figure 4:**
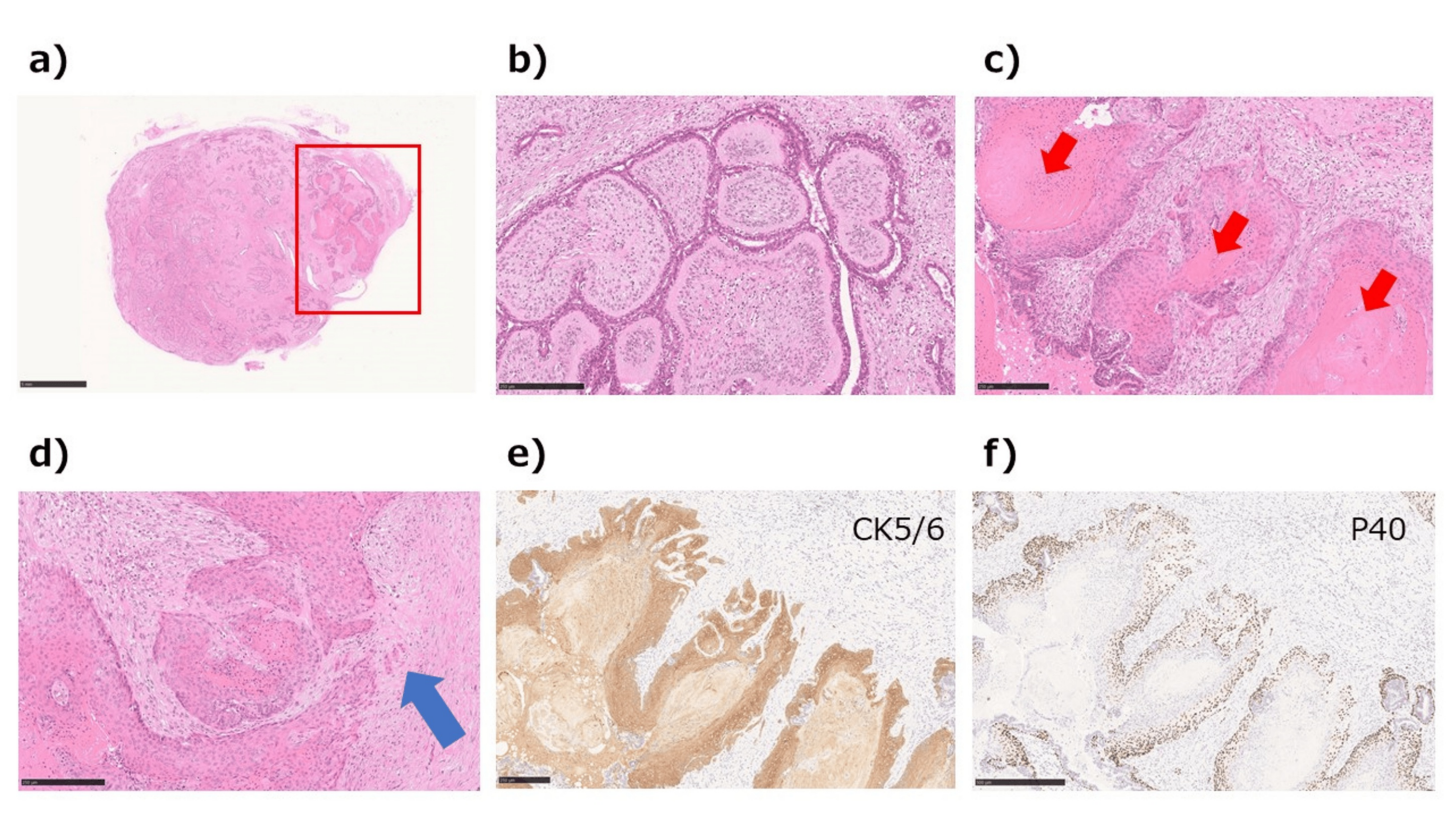
Results of pathological examination of the surgical specimen. (a) The mass is 35 × 27 ｍｍ. SCC is observed in the area delineated by the red box, bar=5 mm. (ｂ) The majority of the mass comprises benign phyllodes tumors, bar=250 μｍ. (ｃ) Squamous epithelium grows in a follicular fashion, filling the lumen with keratinous material (red arrows) in approximately 10% of the total area (red box in a), bar=250 μｍ. (ｄ) The findings are of highly differentiated SCC with intrastromal invasion (blue arrow), bar=250 μｍ. (e) Immunostaining is positive for CK5/6. CK5/6 is a marker for SCC. bar=250 μｍ.  (f) Immunostaining is positive for p40. p40 is a marker for SCC. bar=500 μｍ.

**Figure 5:**
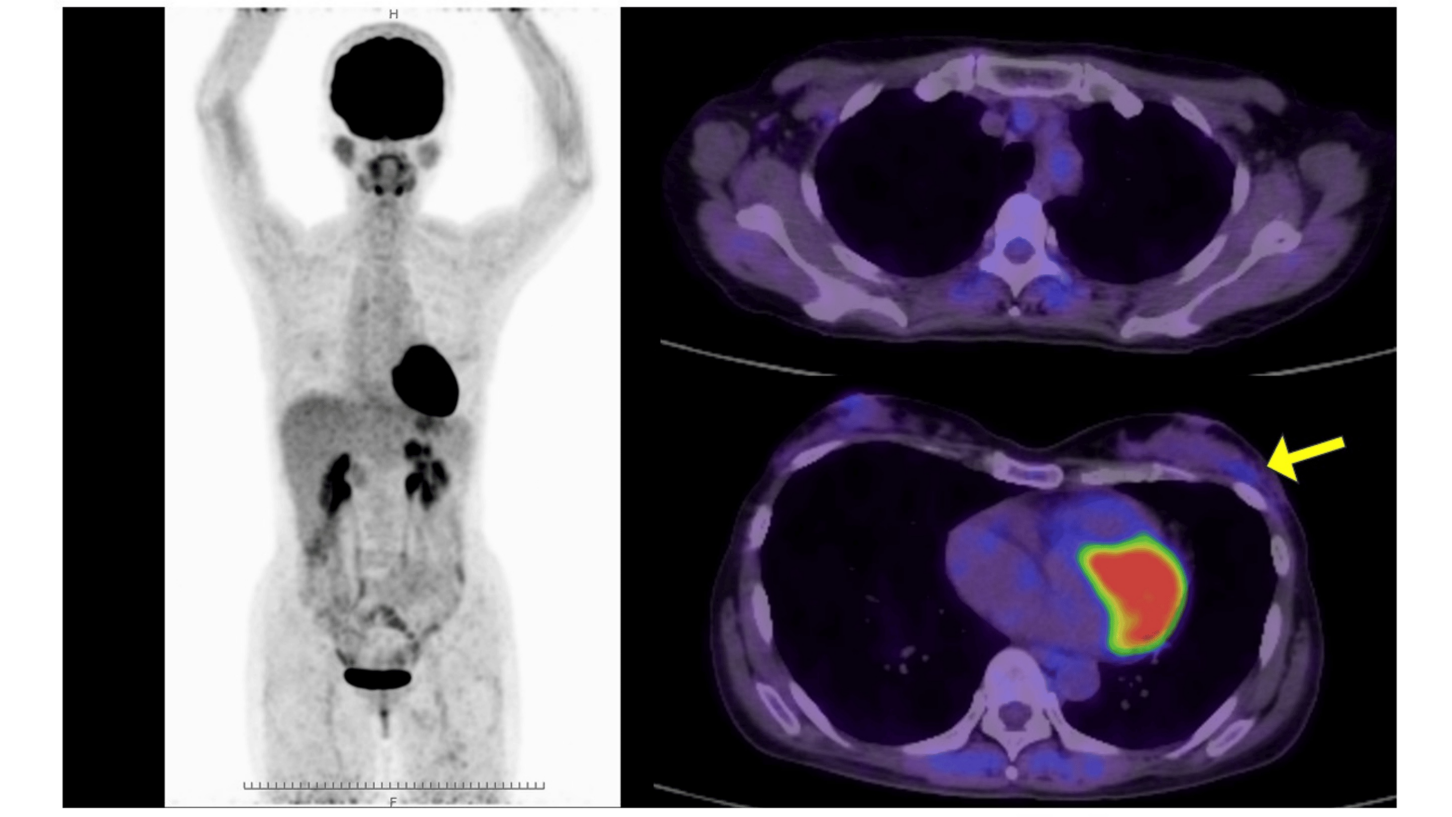
18F-FDG-PET/CT imaging after tumorectomy. ^18^F-FDG-PET/CT imaging after tumorectomy shows no abnormal 18F-FDG uptake throughout the body (a, b) except for slight accumulation (SUV max 1.6) in the left breast, which appears to be postoperative change (c, yellow arrow).

A 49-year-old woman visited her doctor in July 2021 after noticing a mass in her left breast. Screening breast ultrasonography performed in November 2020 showed no abnormalities in the bilateral breasts. Initial breast ultrasonography revealed a 30-mm oval mass with a well-defined border and mild lobulation in the outer left breast. Due to the size of the mass and its appearance only nine months after the screening ultrasonography, the patient was referred to our hospital for further investigation. Physical examination revealed a palpable 30mm mass with good mobility in the outer left breast. Mammography showed a well-defined hyperdense mass in the upper outer left breast (Figure 1). Magnetic resonance imaging (MRI) showed a well-defined 27-mm oval mass in the left breast that exhibited contrast enhancement with a heterogeneous fast-persistent pattern, which was suspected to be a fibroadenoma (Figure 2). Ultrasonography revealed a 26-mm well-defined hypoechoic mass in the upper outer area of the left breast. The mass had an internal blood flow, and elastography showed decreased strain (Figure 3). An ultrasound-guided biopsy of the mass yielded a diagnosis of fibroadenoma. Due to the rapid increase in the size of the mass in a short period of time, a left tumorectomy was performed in September 2021. The pathological diagnosis was of well-differentiated SCC (Estrogen Receptor: Allred score 0, Progesterone Receptor: Allred score 0, Human Epidermal Growth Factor Receptor-2 negative, Ki-67 21%) with 9mm stromal invasion within a benign phyllodes tumor with negative margins. An additional lesion on the margin of the resected phyllodes tumor was suspicious for lobular carcinoma in situ (LCIS) or ductal carcinoma in situ (DCIS), but continuity between the SCC and this lesion could not be confirmed (Figure 4). 18F-fluorodeoxyglucose positron emission tomography-computed tomography (18F-FDG-PET/CT) performed after tumor resection found no abnormal accumulation, ruling out metastasis to the breast from other organs (Figure 5). The tumor was staged as T1bN0M0 Stage I. The patient underwent partial left mastectomy (additional excision) and sentinel lymph node biopsy as additional surgical treatment and received four courses of an AC (doxorubicin + cyclophosphamide) regimen and four courses of docetaxel regimen as adjuvant chemotherapy postoperatively. She then underwent residual breast irradiation (50 Gy/25 Fr). The patient is currently under observation and has had no recurrence four years after surgery.

## Discussion

Primary SCC of the breast is classified as a metaplastic carcinoma, which is defined by the World Health Organization as "a breast carcinoma composed entirely of metaplastic squamous cells that can be keratinizing, non-keratinizing, or tapered and not derived from overlying skin or represents metastasis" [3]. Rosen et al. defined primary SCC of the breast as that in which SCC comprises more than 90% of the cancer lesion, as in the present case [4]. Primary SCC of the breast can be further classified into two types: pure SCC and mixed type, in which adenocarcinoma is also present. The tumor of our patient was classified as pure type, which occurs in 0.046% of cases according to Eggers et al. [5].

In contrast, phyllodes tumors are characterized by the proliferation of epithelial and stromal connective tissue components, and the frequency of ipsilateral simultaneous occurrence of phyllodes tumors and breast cancer is 1%-2%. Breast cancer occurring within a phyllodes tumor is even rarer [6]. Further, the occurrence of SCC, which itself occurs infrequently inside a phyllodes tumor, is extremely rare. A literature search revealed only four case reports in English of squamous cell carcinoma occurring within a phyllodes tumor [6-9].

Most reported cases of SCC are mixed with adenocarcinoma, and it is generally considered that the adenocarcinoma cells metamorphose into SCC cells. However, the pathogenesis of pure-type SCC without an adenocarcinoma component, as in our case, is still unclear [1,10,11].

In the present case, pathological examination revealed an SCC filled with keratinous material in part of a benign phyllodes tumor. Despite the presence of a lesion suspicious of LCIS or DCIS at the margin of the phyllodes tumor, there was no invasive ductal carcinoma in the tumor, and there was no continuity between the lesion at the margin and the SCC. Therefore, it is unlikely that the adenocarcinoma had differentiated into an SCC. The 18F-FDG-PET/CT findings ruled out metastasis of SCC to the breast from other organs.

Yoshida et al. reported a case of pure-type SCC arising within a fibroadenoma, similar to our case. They considered that squamous metaplasia had arisen from myoepithelium within the fibroadenoma [11]. Squamous metaplasia itself is a cellular adaptation to inflammation and other stimuli. This phenomenon has been observed in normal ductal epithelium, papillomas, mastopathy, fibroadenomas, and phyllodes tumors other than malignant tumors [1]. Although squamous metaplasia is rare in phyllode tumors, there have been multiple reports, including oral presentations at academic conferences. In our case, it is reasonable to consider that a benign phyllodes tumor developed squamous metaplasia that finally changed to SCC.

In terms of clinical prognosis, SCC of the breast is widely reported to have a poor prognosis due to rapid growth. However, Eggers et al. analyzed reports of SCC of the breast and found that cases of SCC with spindle cell transformation tended to have a worse prognosis, whereas the prognosis of those without spindle cell transformation did not differ from that of more common types of breast cancer [5]. The treatment of SCC without spindle cell transformation is controversial, but the same treatment for the more common types of breast cancer is usually selected. No spindle cell transformation was observed in the present case, and the patient had a favorable outcome due to early intervention that included surgery, radiation therapy, and chemotherapy.

## Conclusions

We experienced an extremely rare case of SCC arising within a benign phyllodes tumor. No spindle cell transformation was observed in our case, and the patient had a favorable outcome due to early intervention that included surgery, radiation therapy, and chemotherapy.

The preoperative biopsy, in this case, led to a diagnosis of fibroadenoma, and the disease would have surely progressed if the patient was only followed up. Since a biopsy can only evaluate a part of the breast mass, surgical excision should be considered if the mass grows rapidly.
